# Ethnomedicinal Applications, Phytochemistry, and Pharmacological Properties of *Zanthoxylum caribaeum* Lam.: A Comprehensive Review

**DOI:** 10.3390/metabo15090614

**Published:** 2025-09-16

**Authors:** Ahissan Innocent Adou, Ebed Fleurima, Valendy Thesnor, Ander Urrutia, Alain Fournet, Marie-Noëlle Sylvestre, Muriel Sylvestre, Zohra Benfodda, Gerardo Cebrián-Torrejón

**Affiliations:** 1 Laboratoire COVACHIM-M2E, Faculté des Sciences Exactes et Naturelles, Université des Antilles, 97159 Pointe-à-Pitre, France; ahissan.adou@univ-antilles.fr (A.I.A.); valendy.thesnor@ueh.edu.ht (V.T.);; 2 Institut de Systématique, Evolution, Biodiversité (ISYEB), Muséum National d’Histoire Naturelle, CNRS, Faculté des Sciences Exactes et Naturelles, B.P. 592, 97159 Pointe-à-Pitre, France; 3 UPR CHROME, Université de Nîmes, CEDEX 1, 30021 Nimes, France; zohra.benfodda@unimes.fr; 4 BOREA, MNHN-CNRS-SU-IRD-UCN-UA, Laboratoire Biologie Marine, Pointe-à-Faculté des Sciences Exactes et Naturelles Pitre, Université des, B.P. 592, 97159 Pointe-à-Pitre, France; 5 IRD, Laboratoire de Pharmacognosie, Faculté de Pharmacie, Université Paris-Orsay, 92290 Châtenay-Malabry, France

**Keywords:** *Zanthoxylum caribaeum*, rutaceae, natural products, ethnopharmacology, metabolomics

## Abstract

*Zanthoxylum caribaeum* Lam. is a member of the Rutaceae Family that can be naturally found in South and central America (Brazil, Paraguay, Bolivia, Caribbean, etc.). Its traditional medicinal uses are well documented among native communities, such as that of the Guarani, in Paraguay. More than 60 metabolites, including alkaloids, terpenoids, coumarins, and alkylamides, have been identified in its leaves, bark, and fruits. The biological activities and mechanisms of action of several of these compounds, as well as those of crude extracts, have also been investigated by previous studies. As a medicinal and edible plant, *Z. caribaeum* shows promising applications in the pharmacological industry. For the last 25 years, a significant amount of research has been conducted with *Z. caribaeum* to better understand its toxicity and complex mechanisms of action, bringing science-based clinical safety to its traditional uses. This review integrates available knowledge chemical and biological data on this species. It emphasizes the diversity of bioactive metabolites, their associated bioactivities, and provides an updated overview of the plant’s advances in ethnopharmacology, phytochemistry, pharmacology, agricultural exploitation, and potential utilization.

## 1. Introduction

The Rutaceae is a large Family of flowering plants containing at least 150 genera and 1500–2100 species that grow naturally in tropical and subtropical regions [1,2]. This large and diverse Family includes trees, shrubs and aromatic herbs [3]. Perennial grasses and herbaceous lianas are also amongst those plants grouped within this taxon, which is characterized (with exceptions) by thorny branches. Another characteristic of the Family is the presence of aromatic glands that are visible to the naked eye, particularly on young shoots bearing leaves, inflorescences, fruits and cotyledons. Per contra, the stipules are generally absent and in some cases reduced to simple outgrowths [4]. The Rutaceae Family is taxonomically divided into four subfamilies: Rutoideae, Toddaloideae, Rhabdodendroideae and Aurantioindeae. The Rutoideae subfamily is, in turn, divided into five clades; amongst them is the Zanthoxyleae clade, which includes the genus *Zanthoxylum* [5,6]. This genus’ name is derived from the Greek “*xanthon-xylon*” (yellow wood), in reference to the yellowish coloration of its bark, whose fragrance is characteristic and in some cases spicy [7]. The genus *Zanthoxylum* is the second most species-rich genus in the Rutaceae family, comprising over 250 species, mainly distributed across tropical and temperate zones of the southern hemisphere, including regions of America, Africa, Asia and Australia [8,9,10]. Plants within this genus are monoecious or dioecious trees and shrubs easily identifiable by their pinnate leaves, as well as their spine-covered stems, buds and branches. Leaves are alternate and compound, often with odd numbers (3 to 11). Its fruits contain shiny black seeds encased by persistent funicles, and the stems bear a thorny outer bark, a cracked toxic surface, and a central sap layer [11]. The trunk and branches are, like those of its congenera, covered by an outer thorny layer, cracked and toxic bark and a central part, the sap. Flowers are of a greenish/yellowish color and polygamous [12]. In reference to its phytochemistry, the secondary metabolites isolated from parts of *Z. caribaeum* species have demonstrated several pharmacological uses including antioxidant, antimicrobial and antiproliferative activities. In this review, we thoroughly examine all advancements related to the traditional uses, origins, phytochemistry, and pharmacology related to this species, to establish a robust scientific basis for further enhancing the value of this tropical resource.

## 2. Nomenclature and Taxonomy

The nomenclature and taxonomy of *Zanthoxylum* have long been sources of confusion, particularly concerning its relationship with the genus *Fagara* (Table 1). Initially described by Linnaeus in 1757, Brizicky’s study in 1962 suggested that the “simple” perianth of *Zanthoxylum* might have evolved as a secondary state, possibly derived from reductions in the sepals observed in *Fagara* species. The botanist later proposed that *Fagara* should be considered a subset within the broader genus *Zanthoxylum* [13,14,15,16].This view was further supported in 1966 by Hartley [16]. In 1976, Fish et al. [17] confirmed this relationship after analyzing type specimens and transitional forms of both *Fagara* and *Zanthoxylum*. In 1981, Zepernick and Timler [18] formally established “*Zanthoxylum*” as the accepted name in the International Index of Plant Names, despite ongoing use of “*Fagara*” by some authors, particularly in Latin America [19]. Thus, the actual taxonomy of *Zanthoxylum*, is the following: Class Equisetopsida C. Agardh, 1825, Order Sapindales Juss. ex Bercht. & J.Presl, 1820, and Family Rutaceae Juss., 1789. Within this framework, the organism used in this study is categorized as *Zanthoxylum caribaeum* Lam., 1786 falling under the genus *Zanthoxylum* L., 1753 [20]. Table 1 provides a summary of the various synonyms that might be found in the literature.

## 3. Traditional Uses of *Zanthoxylum caribaeum* in Medicine

The bark of *Zanthoxylum caribaeum* is traditionally used for its medicinal properties, including its antimalarial, emmenagogue (regulating the menstrual cycle), and antirheumatic effects. Additionally, the leaves and stem bark are utilized in various forms such as maceration, powder, or decoction to treat diseases like asthma, spasms, fever, herpes, and skin ulcers [23]. The bark is also employed for treating fungal skin infections [24] and has demonstrated antiproliferative properties against certain human cancer cells. In Cuba, it is used for managing asthma, ulcers, rheumatism, and inflammation-related ear-aches [25]. The leaves of *Z. caribaeum* are also orally administered as macerations or decoctions to alleviate stomach ailments and skin diseases [26,27]. In Mexico and tropical America, *Z. caribaeum* is also highly valued for its numerous medicinal uses [27]. The ethnobotanical applications of *Z. caribaeum* across the globe are summarized in Table 2.

## 4. Germination Challenges and Transplanting Strategies for *Z. caribaeum*

Currently, the distribution and abundance of wild *Z. caribaeum* populations have been drastically diminished by uncontrolled harvesting by local populations for its medicinal uses [30].This is significant, given the general lack of interest in it as working wood or firewood due to its thorny structure and intense odor when burned [30]. Additionally, *Z. caribaeum* is not easy to cultivate, with a very low germination rate in the natural environment [22]. In controlled conditions, the substrates producing the best yields were washed sand supplied with a commercial fertilizer, resulting in germination rates of 6.5% and 5%, respectively [22]. Other substrates yielding lower germination rates are the following: bovine manure: 4.5%; mulch: 2%; and sawdust: 1.5% [22]. According to germination rates, seedling emergence is notably slow, typically requiring 4 to 5 months post-sowing. The optimal transplanting period appears to fall between spring and early summer, which may coincide with favorable climatic and edaphic conditions for seedling establishment [22]. Finally, the germination rate appears to be also controlled by temperature, with seeds stored in cold (4 °C) presenting higher germination rates than those stored at room temperature [22].

## 5. Phytochemical Constituents of *Z. caribaeum*

**Figure 1:**
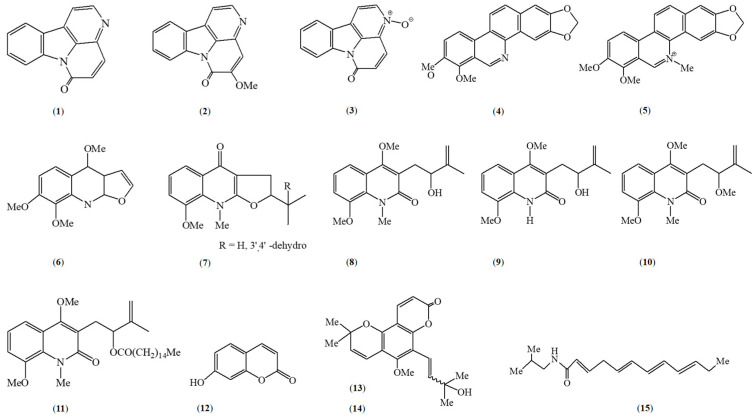
Chemical structures of non-volatile compounds isolated from *Zanthoxylum caribaeum*: alkaloids, coumarins, and alkylamides. Canthin-6-one (**1**), 5-methoxycanthin-6-one (**2**), canthin-6-one-*N*-oxide (**3**), norchelerythrine (**4**), chelerythrine (**5**), skimmianine (**6**), neoacutifoline (**7**), acutifoline (**8**), acutifolidine (**9**), O-methylacutifolidine (**10**), acutifolifine palmitate (**11**), umbelliforine (**12**), cis-avicennol (**13**), trans-avicennol (**14**), sanshool (**15**).

**Figure 2:**
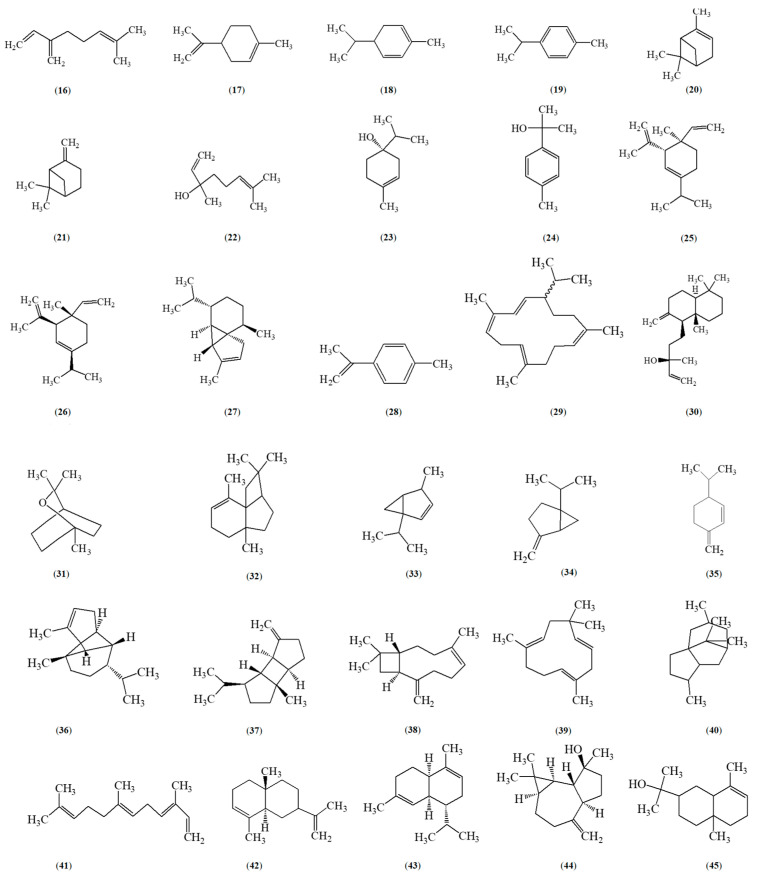

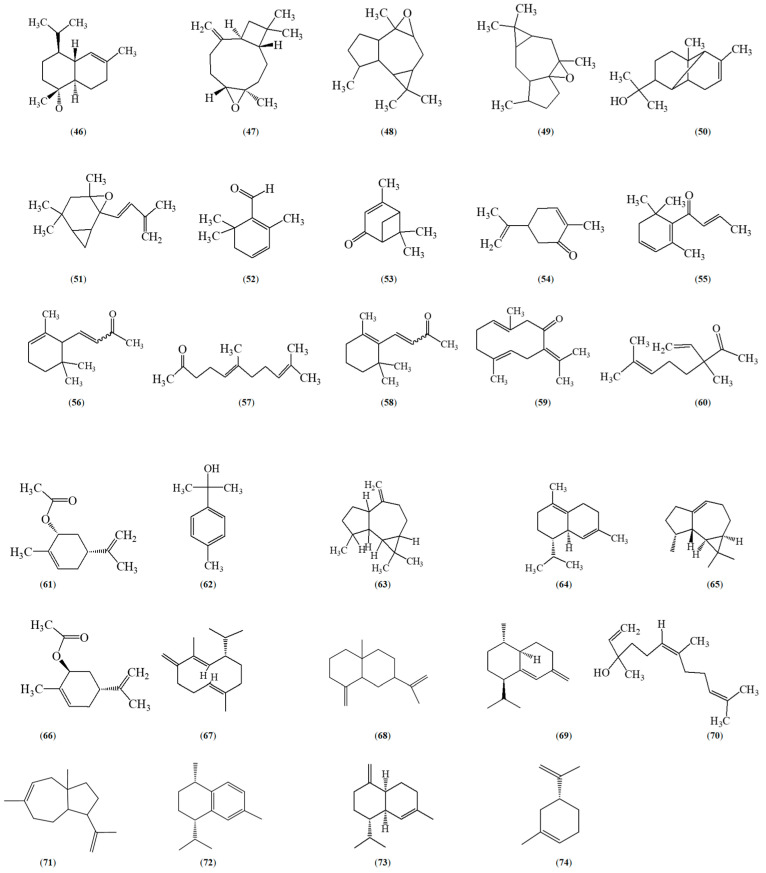
Chemical structures of volatile compounds isolated from *Zanthoxylum caribaeum*: terpenes, monoterpenes, sesquiterpernes, terpenoids and other compounds. myrcene (**16**), limonene (**17**), phellandrene (**18**), cymene (**19**), pinene (**20,21**), linalool (**22**), terpinen-4-ol (**23**), cymen-8-ol (**24**), elemene (**25**), elemene (**26**), cubebene (**27**), cymenene (**28**), thunbergene (**29**), manool (**30**), eucalyptol (**31**), panasinsene (**32**), thujene (**33**), sabinene (**34**), phellandrene (**35**), copaene (**36**), bourbonene (**37**), caryophyllene (**38**), humulene (**39**), patchoulane (**40**), farnesene (**41**), selinene (**42**), muurolene (**43**), spathulenol (**44**), 3-eudesmen-11-ol (**45**), cadinol (**46**), caryophyllene-oxide (**47**), isoaromadendrene epoxide (**48**), ledene-oxide (**49**), copaen-11-ol **(50),** 4,6,6-trimethyl-2-(3-methylbuta-1,3-dienyl)-3-oxatricyclo [5.1.0.0(2,4)]octane (**51**), safranal (**52**), *cis*-verbenone (**53**), carvone (**54**), dasmascenone (**55**), ionone (**56**), geranyl acetone (**57**), ionone (**58**), germacrone (**59**), linalylformate (**60**), cis-carvyl acetate (**61**), terpineol (**62**), aromadendrene (**63**), cadinene (**64**), viridiflorene (**65**), trans-carvyl acetate (**66**), germacrene D (**67**), muurolene (**68**), muurola-4(14),5-diene-trans (**69**), cis-nerolidol (**70**), isodaucene (**71**), calamenene (**72**), muurola-4(14),5-diene-trans(**73**), sylvestrene (**74**).

*Zanthoxylum caribaeum* is noted for its rich array of secondary metabolites across different parts of the plant. For instance, β-carboline alkaloids, canthinone alkaloids, like canthin−6-one (**1**) and 5-methoxycanthin-6-one (**2**) are the main chemotaxonomic characteristic metabolites (Figure 1). Phytochemical investigations have highlighted a variety of compounds including alkaloids (as canthinones), coumarins, and alkylamides that can be sourced from the plant’s diverse tissues [21,24,31,32,33]. Phytochemical screening of *Z. caribaeum* leaves has identified steroids, flavones, flavonols, saponins, tannins, triterpenoids, and xanthones in both organic and aqueous extracts (Table 3) [34]. Furthermore, essential oils (Figure 2) derived from *Z. caribaeum* have been found to contain terpenoids, in various concentrations depending on the geographic regions they are collected in [35,36]. Recent research by Farouil et al. (2022) used advanced techniques like two-dimensional gas chromatography coupled with time-of-flight mass spectrometry (GC × GC-TOFMS) to analyze volatile organic compounds (VOCs) in *Z. caribaeum’s* essential oils from the Guadeloupe archipelago (FWI) [35]. The study revealed a similar metabolomic between *Z. caribaeum* sampled in Guadeloupe and its counterparts from South America (Paraguay). Over thirty terpenoids were identified (Figure 2) [35]. In Paraguay, essential oils from *Z. caribaeum* predominantly contained sesquiterpenes such as cis-nerolidol (**64**) (71.0%), spathulenol (**43**) (3.5%), caryophyllene oxide (**46**) (2.0%), and β-elemene (**66**) (1.9%) [36], constituting about 80% of the total oil composition. Compounds extracted from the essential oil of *Z. caribaeum* species harvested in Brazil were analyzed by GC-MS and identified 20 constituents, with terpenes representing 75.46% of the total composition of the essential oil. The main compounds identified were α-panasinsene (**31**) (12.75%), viridiflorene (**75**) (11.23%), β-elemene (**66**) (10.61%) and β-selinene (**69**) (8.72%); all the others were sesquiterpenes with the exception of one diterpene (Figure 2). Further studies were carried out by Souza et al., (2019) to determine the chemical composition of *Z. caribaeum* essential oil. They demonstrated by GC-MS analysis that the essential oil is essentially composed of terpenes, which account for 63% of the total composition. It is also composed of germacreme-D (68) (20.77%), α-panasinsene (31) (14.40%) and β-selinene (69) (16.68%). Nooreen’s comprehensive review of the genus’s phytochemical composition extensively covers many species but overlooks *Z. caribaeum*, an endemic species of the French West Indies (FWI) [37].

**Table 3:** Phytochemical screening of aqueous and organic extracts of *Z. caribaeum* leaves [34].

Metabolite Family	Extracting Solvent ^a^						
	Me_2_CO	AcOEt	EtOH	Hex	MeOH	CH_2_Cl_2_	Aq
Alkaloids	−	−	−	−	−	−	−
Anthocyanidins	−	−	−	−	−	−	−
Aurones	−	−	−	−	−	−	−
Chalcones	−	−	−	−	−	−	−
Coumarins	−	−	−	−	−	−	−
Steroids	+	+	+	+	+	++	−
Flavones	+	+	+	+	+	+	−
Flavonols	+	+	+	+	+	+	+
Saponins	−	−	−	−	−	−	+
Condensed tannins	−	−	+	−	++	−	+
Triterpenoids	+	+	+	+	+	+	+
Xanthones	+	+	+	+	+	+	+

^a^ Acetone (Me_2_CO), ethyl acetate (AcOEt), ethanol (EtOH), hexane (Hex), methanol (MeOH), dichloromethane (CH_2_Cl_2_), and aqueous (Aq); (+) positive reaction, (++) strong positive reaction and (−) negative reaction.

## 6. Exploring the Bioactive Properties of *Z. Caribaeum*

The biological activities of plants are largely influenced by the presence and composition of secondary metabolites. These organic compounds, produced by plants for various physiological needs such as defense against herbivores, fighting diseases, or attracting pollinators, play a crucial role in their biology and interaction with the environment [38,39]. *Z. caribaeum* is known for harboring a diverse range of secondary bioactive metabolites across its different plant parts, as we presented previously (Table 4).

**Table 4:** Biological activities of metabolites from *Z. caribaeum*.

Compounds	Family	Biological Activities	Plant Part	Reference
		Leishmanicidal activity	Stem bark	[32]
Canthin-6-one (**1**)	Alkaloid	Trypanosomalactivity	Stem bark	[28]
		Antifungal activity	Stem bark	[40]
		Leishmanicidal activity	Stem bark	[32]
5-methoxycanthin-6-one (**2**)	Alkaloid	Trypanosomal activity	Stem bark	[28]
		Antifungal activity	Stem bark	[40]
Canthin-6-one-N- oxyde (**3**)	Alkaloid	Trypanosomal activity	Stem bark	[28]
Chelerythrine (**5**)	Quinolone Alkaloid	Not specified	Root	[21,33]
Skimmianine (**6**)	Quinolone Alkaloid	Not specified	Root	[21,41]
Neoacutifolidine (**7**)	Quinolone Alkaloid	Not specified	Leaves	[31]
Acutifoline (**8**)	Quinolone Alkaloid	Not specified	Leaves	[31]
Acutifolidine (**9**)	Quinolone Alkaloid	Not specified	Leaves	[31]
O-methylacutifoline (**10**)	Quinolone Alkaloid	Not specified	Leaves	[31]
Acutifolidine palmitate (**11**)	Quinolone Alkaloid	Not specified	Leaves	[31]
Umbelliforine (**12**)	Coumarin	Antiproliferative activity	Root bark	[18]
Trans-avicennol (**14**)	Coumarin	Antiproliferative activity	Root bark	[29]

### 6.1. Antioxidant Activities of Z. caribaeum

The antioxidant properties of the essential oil from the leaves of *Z. caribaeum* were analyzed using the 2,2-diphenyl-1-picrylhydrazyl (DPPH) free radical scavenging method. The essential oil and the synthetic antioxidant BHT (butylated hydroxytoluene) had sequestration percentages of 90.22% and 94.5%, respectively. Moreover, their half-maximal inhibitory concentration (IC_50_) was 1.50 and 2.77 µg/mL, respectively. This study demonstrated that the essential oil has an activity similar to that of the synthetic molecule BHT [42]. This activity may be linked to certain terpenoids such as monoterpenoids and sesquiterpenoids [43]. Souza et al. also evaluated the antioxidant potential of various organic extracts from *Z. caribaeum* leaves. The authors were able to demonstrate that the ethanolic extract had the highest percentage of free radical scavenging (71.2%) and a low median inhibitory concentration (IC_50_ = 24.39 μg/mL), which is the amount of antioxidant substance needed to reduce the initial DPPH concentration by 50%. These different values reveal the antioxidant potential of this extract [34] (Table 5). In another study, Shahidi et al. explain that the tannins and flavonoids identified in polar extracts are free radical scavengers [44].

**Table 5:** Antioxidant activity of aqueous and organic solvent extracts from the leaves of *Z. caribaeum* by the DPPH method [34].

Extracting Solvent	Test Solution ^a^	% Capture DPPH ^b^	IC_50_ (μg/mL) ^c^
	EtOH	71. 12	24.39
	Me_2_CO	66.16	29.67
	MeOH	61.99	27.98
	A_C_OEt	48.56	29.67
	BHT (positive control)	92.80	7.93

^a^ Ethanol (E_t_OH), acetone (Me_2_CO), methanol (MeOH), ethyl acetate (A_C_OEt) and BHT (commercial synthetic antioxidant Butylhydroxytoluene). ^b^ Percentage of radical sequestration (DPPH) at 200 µg/mL (2,2-diphenyl-2-picrylhydrazyl); ^c^ Concentration of *Z. caribaeum* leaves extract is necessary to reduce 50% of the DPPH radicals.

### 6.2. Leishmanicidal Activity of Z. caribaeum

In 2002, Ferreira et al. evaluated the leishmanicidal *in vitro* activity of crude extracts of *Z. caribaeum* stem bark using three different Leishmania species. These crude extracts exhibited activity at 100 µg/mL. They also tested the activity of canthin-6-one (**1**) and 5-methoxycanthin-6-one (**2**), two alkaloids isolated from the chloroform extract of *Z. caribaeum* stem bark, using an in vivo test and in infected mice. Intralesional administration of 10 mg/kg canthin-6-one (**1**) for 4 days did not significantly reduce the parasite load [32]. Table 6 summarizes the in vitro activity of crude extracts of *Z. caribaeum*, canthin-6-one (**1**) and 5-methoxy canthin-6-one (**2**) against three strains of promastigote forms of *Leishmania* spp.

Serna et al. [45] conducted studies on the leishmanicidal activity in an in vivo test of *Z. caribaeum* leaves in mice infected using oral and intralesional treatment. Intralesional administration at a dose of 50 mg/kg reduced the parasite load by 72%. Oral administration at doses of 50 mg/mL and 10 mg/mL resulted in reductions of 50% and 55%, respectively.

### 6.3. Antichagasic Effects of Z. caribaeum Against Trypanosoma cruzi

According to the World Health Organization’s (WHO) estimates in 2022, 6 to 7 million people were infected worldwide with *Trypanosoma cruzi.* This parasite is responsible for the Chagas disease or American trypanosomiasis [46], with the majority of infections occurring in Latin America. Ferreira et al. evaluated the trypanocidal activities of total alkaloid extracts from the stem bark of *Z. caribaeum*, canthin-6-one (**1**), 5-methoxycanthin-6-one (**2**), and canthin-6-one *N*-oxide (**3**) using an in vivo test and oral or subcutaneous treatment for 2 weeks in mice with acute or chronic infection. Administration of a dose of 5 mg/kg/day of canthin-6-one (**1**) derivatives and 50 mg/kg/day of total alkaloid extracts had a limited effect on acute and chronic infection. However, a dose of 5 mg/kg/day of canthin-6-one (**1**) had a significant effect on acute and chronic infection (Table 7) [28].

Ferreira et al. examined the trypanocidal activity of extracts from *Z. caribaeum* and two compounds identified in these extracts, namely canthin-6-one (**1**) and 5-methoxycanthin-6-one (**2**), against trypomastigote and amastigote forms through in vitro and in vivo testing. Treatment was administered orally or subcutaneously for two weeks in acutely infected mice.

In the in vitro test against the trypomastigote form, they found that the ethanolic leaf extract and canthin-6-one (**1**) produced the best results. The values obtained are close to those of the reference drug (benznidazole). Thus, at doses of 250 μg/mL, the ethanolic extract and canthin-6-one (**1**) reduced the trypomastigote form by 78% and 79%, respectively, compared to 87% for the reference drug (benznidazole).

Canthin-6-one (**1**) and 5-methoxycanthin-6-one (**2**) showed activity against the amastigote form of 90% and 66.4%, respectively, at doses of 15.1 μm. The activities of these two molecules are close to that of the benznidazole, which is 97.5% at doses of 192 μm.

For the in vivo test, the authors showed that, at doses of 10 mg/kg, the ethanolic extract of *Z. caribaeum* leaves significantly reduced parasitic infection [47].

Bilbao et al. also examined the trypanocidal activity of chloroform extracts from *Z. caribaeum* seedlings against trypomastigote forms in an in vitro test in infected mice. They showed that the dichloromethane extract from 24-month-old plants had the best activity, with a 77% reduction in parasites at doses of 250 μg/mL and an IC_50_ of 71 μg/mL [48].

### 6.4. Antimicrobial Activities of Z. caribaeum

The dichloromethane extract from the bark of *Z. caribaeum* stems showed interesting antifungal activity against *Candida albicans*, *Aspergillus fumigatus*, and *Trichophyton mentagrophytes*. Bioguided purification of this extract allowed two compounds to be isolated: canthin-6-one (**1**) and 5-methoxycanthin-6-one (**2**). An in vitro evaluation of the activity of these two molecules was carried out against 12 microorganisms. Canthin-6-one (**1**) showed good activity against 11 fungi, including inhibition of 12.8 μmol/L on 9 fungi, while 5-methoxycanthin-6-one (**2**) showed weak activity against these 11 fungi [40]. Table 8. Summarizes the antifungal activity of canthin-6-one (**1**) and 5-methoxy-canthin-6-one (**2**) expressed as MIC (minimum inhibitory concentration (μmol/L)), with ketoconazole as the reference drug [40].

The antifungal activity of the ethanolic extract of *Z. caribaeum* bark was demonstrated using the disk diffusion method. This extract showed significant activity at 500 µg/disc against common dermatophytes found in domestic animals [49]. In addition, in vitro testing of acetone extract from *Z. caribaeum* in mice improved skin lesions caused by tinea pedis (athlete’s foot). The therapeutic efficacy of this extract at a dose of 2.5 mg/kg was comparable to 1 mg/kg of clotrimazole (reference drug) [50].

Ortega-Buitrago et al. explored antimicrobial activity using an in vitro test of the ethanolic extract of the leaves and bark of *Z. caribaeum* against four microorganisms: *S. aureus*, *S. mutans*, *E. coli*, and *Morganella morganii*. During their study, they demonstrated that the ethanolic extract of *Z. caribaeum* bark has activity against Gram-positive bacteria *S. aureus* and *S. mutans*, with an inhibition zone of 8 mm. However, the various extracts showed no activity against Gram-negative bacteria *E. coli* and *M. morganii* [51].

In addition, Chaves-Bedoya et al. evaluated the antibacterial activity of ethanol extracts from *Z. caribaeum* leaves against *Burkholderia glumae*, a Gram-negative soil bacterium. At the end of this study, they showed that the ethanol extract from *Z. caribaeum* leaves has no significant biological activity against *B. glumae* [52].

### 6.5. Acaricidal Activity of Z. caribaeum

Nogueira et al. examined the acaricidal activity of *Z. caribaeum* essential oil against *Rhipicephalus microplus*. During this study, they demonstrated that essential oil at a concentration of 5% caused 100% mortality after two days of treatment in engorged females [53].

Souza et al. also examined the acaricidal activity of acetone, ethanol, and methanol extracts from *Z. caribaeum* leaves against *D. gallinae*. They obtained mortality rates of 37.6% and 27.2% for the methanol and ethanol extracts, respectively, at a concentration of 1 g/mL. At the same concentration, the acetone extract resulted in a mortality rate of 25.6% against *D. gallinae* [34].

### 6.6. Antimalarial Activity of Z. caribaeum

Cebriàn-Torrejòn et al. reported the in vitro antimalarial activity of dichloromethane, ethanolic, and methanolic extracts of *Z. caribaeum* stem bark, as well as three compounds isolated from the dichloromethane extract (trans-avicennol (**14**), canthin-6-one (**1**), 5-methoxycanthin-6-one (**2**)) against strains of *P. falciparum* (the parasite responsible for the most severe form of malaria). Four distinct strains were tested: the Colombian chloroquine-resistant FcB1 strain, the Brazilian mildly chloroquine-resistant strain PFB, the multidrug and chloroquine-resistant strain K1 from Thailand, and the chloroquine-sensitive, but mefloquine resistant F32 strain from Tanzanian. The dichloromethane and ethanol extracts demonstrated the most pronounced effects against the K1 and 32 strains, with IC_50_ values of 8.9 μg/mL and 10.5 μg/mL, respectively. Among the isolated compounds, F32 showed the highest sensitivity to avicennol and canthin-6-one, with IC_50_ values of 0.5 and 2.0 µg/mL, respectively [54].

The antimalarial activities measured following [3H]-hypoxanthine incorporation were confirmed [54] (Table 9); it was also reported by the authors that neither trans-avicennol (**14**), canthin-6-one (**1**), 5-methoxycanthin-6-one (**2**) nor the dichloromethane, ethanol and methanol extracts of *Z. caribaeum* had a hemolytic effect on the erythrocytes.

**Table 9:** IC_50_ (μg/mL) of *Z. caribaeum*-isolated compounds and extracts [54].

Compounds	*P. falciparum* Strain IC_50_ (μg/mL)			
	F32	K1	PFB	FcB1
Trans-avicennol (1)	0.5	2.7	1.2	2.2
Canthin-6-one (2)	2.0	5.3	3.2	4.0
5-methoxycanthin-6-one (3)	10.4	5.1	Nt ^b^	Nt
**Extracts**				
DCM	8.9	8.9	Nt	Nt
EtOH	10.5	9.3	Nt	Nt
MeOH	89.5	>100	Nt	Nt
Chloroquine ^a^	2.6	77.4	27.8	62.8

^a^ Chloroquine (Reference drug) concentrations are in nM; ^b^ Nt: not tested.

### 6.7. Anti-Inflammatory Activity of Z. caribaeum

In 2005, Márquez et al. evaluated the in vivo anti-inflammatory activity of the ethanolic extract of *Z. caribaeum* stem bark against inflammation of the mouse ear. At a dose of 2 mg/ear, they obtained a 77.7% inhibition of edema caused by PMA (phorbol myristate acetate) [25].

Villalba et al. also evaluated the anti-inflammatory activity of hexane, ethyl acetate, and ethanol extracts of *Z. caribaeum* leaves against rat paw edema. The inhibition percentages were 57%, 48%, and 43% for hexane, ethyl acetate, and ethanol extracts, respectively, at a dose of 200 mg/kg [55].

### 6.8. Antiproliferative Activity of Z. caribaeum

Cebrián-Torrejòn et al. examined the antiproliferative activity of a methanol extract of *Z. caribaeum* root bark and a pyranocoumarin isolated from this extract, trans-avicennol. (**14**). At a dose of 100 mg/mL, the methanol extract had a maximum activity of 30% on human neural stem cells, while trans-avicennol (**14**) was inactive at a dose of 34.2 μg/mL [29].

### 6.9. Effects Against Cardiovascular Disease

In 2017, Domanech-Carbo et al. showed how canthin-6-one (**1**) modified the activity of soluble guanylate cyclase (sGC) regardless of the presence of SNP (sodium nitroprusside), a NO (nitric oxide) donor [56]. sGC, composed of two subunits in mammals, catalyzes the conversion of guanosine triphosphate (GTP) to cyclic guanosine monophosphate (cGMP) and phosphate. The binding of its main activator, nitric oxide (NO), to the ferrous prosthetic group cleaves the proximal Fe^2+^. Histidine significantly stimulates the enzymatic production of cGMP. To better understand the molecular mechanism of sGC activation and deactivation (based mainly on NO interaction with the active site of the enzyme) for therapeutic intervention targeting pathologies involving the NO-cGMP pathway, the inhibitory effect of cantin-6-one on sGC was studied using electrochemical and bio-chemical methods. The results showed that canthin-6-one (**1**) inhibited basal sGC activity in a dose-dependent manner at 1, 10, 100 and 300 µmol/L. In the absence of canthin-6-one (**1**), 100 µmol/L SNP increased the activity of sGC by eight-fold.

### 6.10. Larvicidal Activity

In 2009, Sanabria et al. conducted biological tests on aqueous extracts of *Z. caribaeum* to verify its larvicidal activity against *Aedes aegypti* larvae. Aqueous plant extracts were prepared at concentrations of 5, 15 and 25% for the larvae. Twenty larvae per group were exposed to the treatments in cups containing 100 mL of distilled water, with positive and negative controls. Each treatment was performed in triplicate and repeated twice. Larval mortality was recorded at 24, 48 and 72 h post-treatment. The results showed that *Z. caribaeum* exhibited no larvicidal activity at a minimum concentration of 5% [57].

## 7. Toxicological Aspects of *Z. caribaeum*

Nogueria et al. conducted studies on the toxicological effects of essential oil from the leaves of *Z. caribaeum* from Brazil on the development of *Rhodnius prolixus* (the main vector of Chagas disease). At doses of 0.5 to 5 μL per insect, topical treatment with the essential oil resulted in paralysis (18.88 to 33.33%) and mortality (80 to 98.9%). Dietary treatment with the essential oil resulted in mortality (48.8 to 100%) and paralysis (2.22 to 7.77%) at doses of 0.5 to 5 μL/mL of blood. Finally, continuous treatment at a dose of 5 μL/cm^2^ resulted in mortality of 63.3% [58].

Cebrián-Torrejòn et al. also tested the toxicity in human fetal cells of dichloromethane and ethanol extracts, as well as canthin-6-one (**1**) and trans-avicennol (**14**), three compounds isolated from the dichloromethane extract of *Z. caribaeum* stem bark, using MRC5 cells (human fetal cells). The results showed that trans-avicennol (**14**) had high toxicity at an IC_50_ of 4.4 μg/mL. Canthin-6-one (**1**) had an IC_50_ of 9.4 μg/mL. The dichloromethane and ethanol extracts showed IC_50_ values of 12.3 and 13 μg/mL, respectively [54].

## 8. Conclusions and Perspectives

*Zanthoxylum caribaeum* is a widely used plant species in the Americas. It is a remarkable source of compounds traditionally used medicine to treat skin infections and digestive disorders, rheumatism, and other illnesses. Almost every part of the plant is used, with specific applications for barks, roots, fruits, and leaves, prepared as decoctions, infusions, macerations or directly as powder. Several compounds have been isolated and characterized from these plant parts, including alkaloids, coumarins and terpenes, which have demonstrated antioxidant, anti-inflammatory, antimalarial and anticancer activities. Although most of the isolated compounds exhibit pharmacological activity, their mechanisms of action remain largely unknown. Therefore, more in-depth studies are required, particularly those that connect traditional uses with confirmed pharmacological effects. While some toxicological data are available, further research into their secondary metabolites is necessary to explore both current and potential therapeutic applications.

## Figures and Tables

**Table 1:** Synonyms of *Zanthoxylum caribaeum* Lam. 1786.

Name	Reference
*Fagara caribaea* (Lam.) Krug&Urb.	[3]
*Fagara* (Martius ex Engler) Engler	[21]
*Fagara chiloperone* var. *angustifolia* (Engl.) Engl. Ex Chodat & Hassl.	[22]
*Zanthoxylum chiloperone* Martius ex Engler	[21]
*Zanthoxylum chiloperone* var. *angustifolium* Engl.	[22]
*Zanthoxylum rugosum A*. St. -Hill. &Tul.	[21]
*Zanthoxylum aromaticum* sensu Duss	[3]

**Table 2:** Global ethnobotanical applications of *Z. caribaeum*.

Part Used	Country	Traditional Applications	References
Root bark	Paraguay, Cuba	Antirheumatic	[25,28]
Stem bark, leaves	Paraguay	Skin diseases	[24,27]
Stem bark	Paraguay	Emmenagogue	[29]
Stem bark	Paraguay	Antimalarial	[29]
Root bark	Paraguay	Anticancer	[29]
Stem bark	Cuba	Antiasthmatic	[25,27]
Stem bark	Cuba	Anti-ulcer	[25,27]
Leaves	Latin America	Stomach ailments	[27]

**Table 6:** *In vitro* activity (μg/mL) of *Z. caribaeum* crude extracts, canthin-6-one (**1**) and 5- methoxy canthin-6-one (**2**) towards three strains of promastigote forms of *Leishmania* spp. [32].

Extract and Compound	*L. braziliensis*	*L. amazonensis*	*L. donovani*
*N*-methylglucamine	>100	>100	>100
Pentamidine ^a^	5	5	5
Alkaloidal extract (CH_2_CL_2_)	100	100	100
Methanolic extract	>100	>100	>100
Canthin-6-one	100	100	100
5-methoxycanthin-6-one	100	100	100

^a^ Reference drug.

**Table 7:** Cure rates in mice presenting *Trypanosoma cruzi* acute infections after a 2-week treatment with benznidazole at 50 mg/kg/day, canthin-6-one (**1**) at 5 mg/kg/day, 5-methoxy-canthin-6-one (**2**), canthin-6-one *N*-oxide (**3**) at 5 mg/kg/day, and crude *Zanthoxylum caribaeum* alkaloid-containing extract at 50 mg/kg/day. The remission of infection was considered in cases of negative parasitaemia [28].

Treatment	*n* ^b^	Negative Parasitaemia/Number of Survivors (Days Post-Infection)				
Post-Infection Time	0 Days	18 Days	32 Days	45 Days	56 Days	68 Days
Control	20	0/19	4/17	6/14	0/12	3/11
Benznidazole	20	14/20	12/20	10/19	11/19	17/19
1 oral	20	5/20	11/20	15/20	17/20	19/20
1 s.c	21	6/21	6/21	12/21	10/21	19/21
2 oral	9	8/9	2/7	1/7	4/7	3/7
2 s.c. ^a^	8	6/8	5/8	3/8	4/8	6/8
3 orally administered	10	0/10	0/7	0/6	6/6	6/6
Crude *Zanthoxylum caribaeum* alkaloid oral	12	1/12	0/10	5/9	5/9	8/9
Crude *Zanthoxylum caribaeum* alkaloid s.c	12	1/11	1/11	10/11	10/11	5/10

^a^ s.c: subcutaneous; ^b^ *n*: number of mice.

**Table 8:** Antifungal activity of canthin-6-one (**1**) and 5-methoxy-canthin-6-one (**2**) expressed as MIC (μmol/L). Ketoconazole as reference drug [40].

Species	Canthin-6-One	5-Methoxycanthin-6-One	Ketoconazole
*Candida albicans*	56.1	190.3	46
*Candida glabrata*	12.8	90.8	5.3
*Candida tropicalis*	12.8	45.4	5.3
*Aspergillus fumigatus*	56.1	97.5	45.9
*Aspergillus niger*	12.8	22.7	5.3
*Aspergillus terreus*	12.8	>181.6	5.3
*Cryptococcus neoformans*	12.8	45.4	5.3
*Geotrichum candidum*	12.8	>181.6	5.3
*Saccharomyces cerevisiae*	12.8	>181.6	5.3
*Trichophyton mentagrophytes* var. *interdigitale*	>216.2	12.3	5.8
*Trichosporon beigelii*	12.8	22.7	5.3
*Trichosporon cutaneum*	12.8	90.8	5.3

## Data Availability

Not applicable.
